# Preliminary Clinical and Functional Outcomes After Combined Treatment of Lateral Osteochondral Lesions of the Talus and Chronic Ankle Instability Using Autologous Minced Cartilage and Retinaculum Flap Augmentation

**DOI:** 10.3390/medicina62061042

**Published:** 2026-05-28

**Authors:** Kajetan Klos, Stefan Landgraeber, Klaus-Edgar Roth, Preslav Penev, Felix Bachelier, Joe Wagener, Philipp Winter

**Affiliations:** 1 Department of Trauma, Hand and Reconstructive Surgery, University Hospital Jena, Am Klinikum 1, 07747 Jena, Germany; 2 Meliva Gelenkzentrum Rhein-Main, Fuß- Und Sprunggelenkschirurgie, Frankfurter Straße 94, 65239 Hochheim, Germany; 3 Department of Orthopaedic and Traumatology, Medical University of Varna, 9002 Varna, Bulgaria; 4 Department of Orthopaedic Surgery, Saarland University, Kirrberger Straße, 66421 Homburg, Germany; 5 Department of Orthopaedic Surgery, Hôpital Kirchberg, L-2540 Luxembourg, Luxembourg

**Keywords:** ankle arthritis, chronic ankle instability, minced cartilage, osteochondral lesion of talus, Skillcourt

## Abstract

***Background*****:** Osteochondral lesions of the lateral talar shoulder (OLT) represent a significant therapeutic challenge, particularly when associated with chronic lateral ankle instability (CAI). While bone marrow stimulation is well established for talar lesions, clinical evidence on the minced cartilage technique (AutoCart™, Arthrex, Naples, FL, USA) in this specific localization remains limited. This study aimed to evaluate the short-term clinical and functional outcomes following combined treatment of lateral OLT and CAI using autologous minced cartilage and open ligament repair. ***Methods*****:** Nine patients (mean age: 39.8 years) with symptomatic lateral OLT and concomitant CAI were treated using the minced cartilage technique in conjunction with lateral ligament reconstruction. The mean defect size was 64.8 ± 30.2 mm^2^. Clinical and functional outcomes were assessed at a mean follow-up of 15.8 months using the Foot Function Index (FFI), Visual Analog Scale (VAS) for pain, return-to-sport status, and a motor-cognitive test (Skillcourt system). Postoperative complications and patient satisfaction were recorded. ***Results*****:** No postoperative complications occurred. All patients reported satisfaction with surgical outcomes. The mean postoperative FFI was 14.92 ± 12.74, indicating minimal functional limitations in daily life. Skillcourt testing revealed no significant side-to-side differences in most tests, except for the Single Leg Stability Test, which showed a significant deficit on the operated side (*p* = 0.027; Cohen’s d = 0.9). Reduced dorsiflexion limited test performance of four patients. The average postoperative VAS during functional testing was 1.42 ± 1.62. ***Conclusions***: Combined treatment of lateral OLT and CAI using the minced cartilage technique and open ligament stabilization yields favorable short-term clinical and functional outcomes. Despite minor limitations in dorsiflexion, patients achieved high satisfaction rates and functional recovery. These preliminary findings support the technical feasibility and short-term clinical applicability of combining biological cartilage repair with mechanical stabilization for lateral talar lesions < 100 mm^2^. However, larger prospective studies with longer follow-ups are warranted to validate these findings.

## 1. Introduction

Symptomatic osteochondral lesions of the talus dome (OLT) represent a real therapeutic challenge. An osteochondral defect (OCD) or a lesion of the talus can be associated with chronic lateral ankle instability (CAI) [1,2]. Without appropriate treatment, these lesions can progress to tibiotalar osteoarthritis [2]. Bone marrow stimulation is an established technique for the treatment of osteochondral lesions of the medial and lateral talar shoulder [3]. In principle, osteochondral lesions of the lateral talar shoulder tend to be smaller than those of the medial talar shoulder [4]. Moreover, the etiology of these injuries differs between the two locations. Osteochondral lesions of the lateral talar shoulder are more frequently attributed to a traumatic origin [5]. This raises the question of whether the current therapeutic recommendations for osteochondral lesions of the talus can be applied universally to both locations without restriction. A promising method of treating osteochondral lesions is the minced cartilage technique. Osteochondral lesions of the lateral talar shoulder are frequently associated with concomitant ligamentous instability. In the context of open ligament reconstruction for chronic ankle instability, the osteochondral lesion can often be visualized without the need for extending the surgical approach. Because osteochondral lesions of the lateral talar shoulder frequently occur in patients with chronic lateral ankle instability, simultaneous treatment of both pathologies during the same surgical procedure may represent an optimal therapeutic strategy. Clinical evidence regarding autologous minced cartilage procedures remains limited. However, preliminary data suggest that minced cartilage is a safe and viable technique for cartilage repair [6]. Published studies have demonstrated satisfactory clinical outcomes, with failure and revision rates comparable to those of other established cartilage repair methods [7,8,9,10]. Nevertheless, further comparative trials are needed to enable direct comparisons with alternative cartilage repair techniques. Additionally, long-term outcome data are essential for assessing the durability of minced cartilage implants and their long-term effectiveness in maintaining joint function [6]. The aim of this study was to evaluate short-term clinical and functional outcomes after combined treatment of lateral osteochondral lesions of the talus and chronic ankle instability using autologous minced cartilage implantation and open ligament repair.

## 2. Materials and Methods

This retrospective study included patients treated between January 2022 and March 2024 for chronic lateral ankle instability with a concomitant lateral osteochondral lesion of the talus. All patients had failed prior conservative treatment. Patients with advanced osteoarthritis, previous cartilage repair procedures, or neurological disorders affecting lower limb function were excluded. Inclusion criteria were age ≥ 18 years, presence of a symptomatic lateral osteochondral lesion of the talar dome, and concomitant chronic lateral ankle instability after failure of conservative treatment.

### 2.1. Surgical Technique

**Figure 1:**
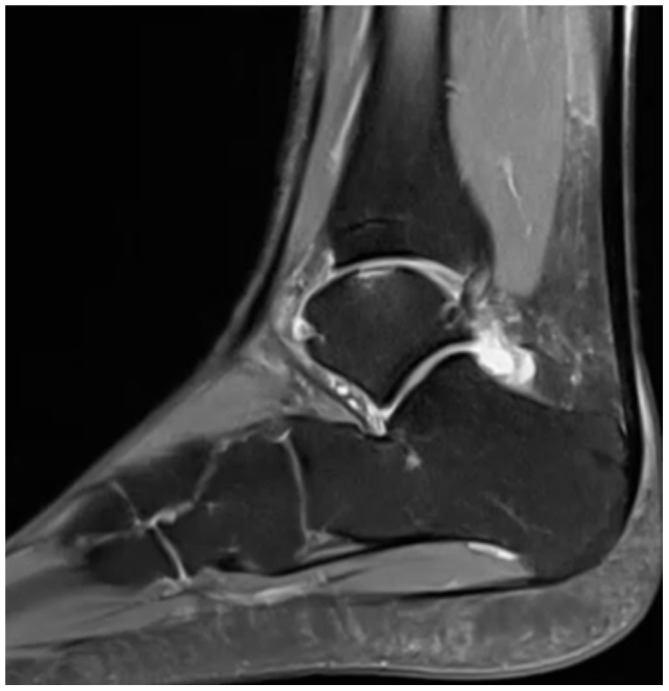
MRI of a lateral osteochondral lesion of the talus.

**Figure 2:**
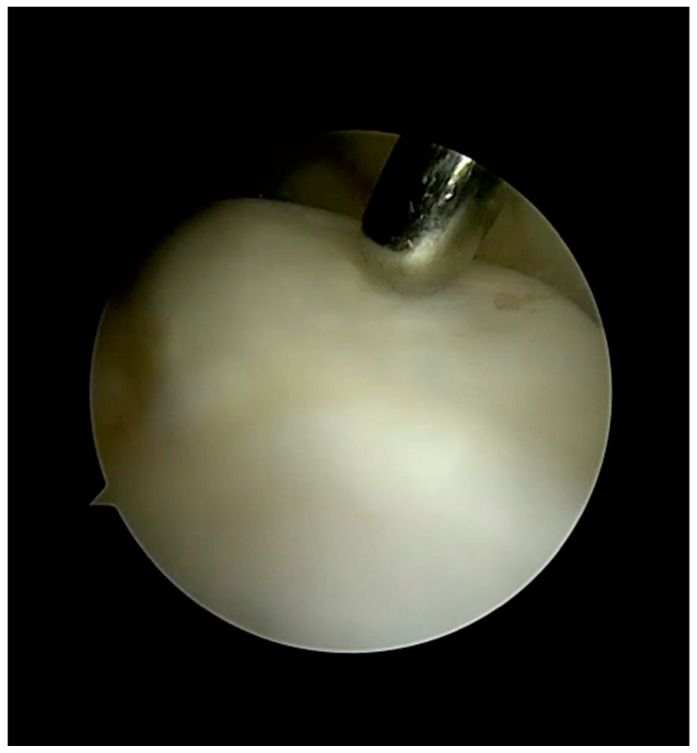
Arthroscopic visualization of the osteochondral lesion of the lateral talus.

**Figure 3:**
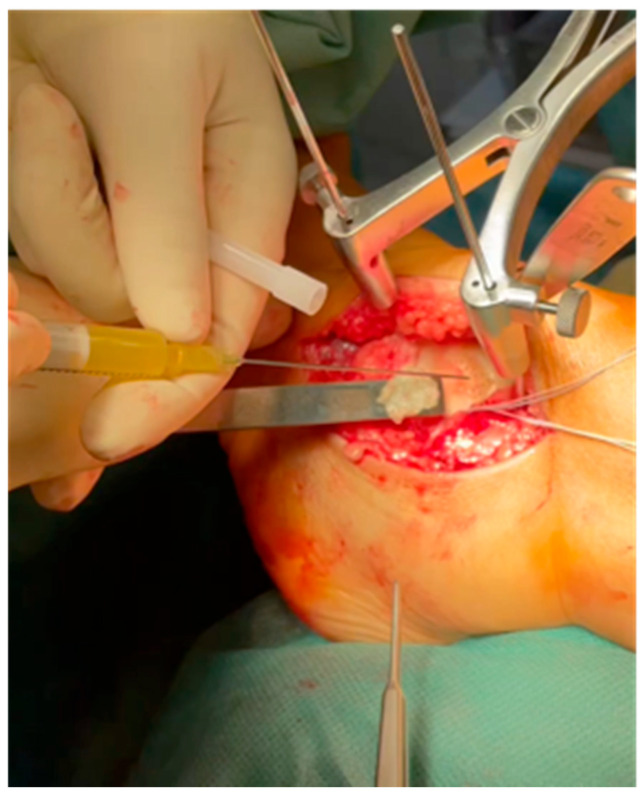
Reconstruction of the osteochondral defect using minced cartilage after previous microfracturing.

The patient was positioned supine on the operating table under general anesthesia. A diagnostic arthroscopy of the ankle joint was performed prior to lateral ligament repair, with concomitant assessment and management of intra-articular pathologies (Figure 1). Intra-articular lesions, including osteochondral defects of the talus, syndesmosis injury, synovitis, and anterior impingement syndrome secondary to osteophyte formation, were identified and documented via arthroscopy (Figure 2), addressed according to intraoperative findings (Table 1). Diagnostic arthroscopy was performed initially to assess the ankle joint, confirm the osteochondral lesion, and address concomitant intra-articular pathologies before proceeding with the open ligament stabilization according to Tourné et al. Upon identification of the talar osteochondral defect (OCD), treatment was determined based on the stage and size of the lesion. A curvilinear incision was made over the lateral malleolus, To treat the osteochondral defect a transligamentous approach was used [11]. Autologous minced cartilage (AutoCart, Arthrex, Naples, FL, USA) was utilized in all cases (Figure 3). The size of the lesions was visually assessed intraoperatively. The cartilage defect is debrided in a standardized manner using a sharp curette. After debridement, the cartilage can be harvested from this location using a 3.0 mm shaver device with a collecting device. If needed, a subchondral bone graft or substitute is impact into the defect. The filling should be leveled with the adjacent subchondral bone. The autologous cartilage fragments are delivered to the defect area using an applicator device to ensure full coverage of the defect. Complete filling to the level of the surrounding cartilage is not required; a filling of approximately 80% to 90% is sufficient. The defect was intentionally not filled completely to the level of the surrounding cartilage in order to avoid graft protrusion into the joint and potential mechanical irritation during the early healing phase. In addition, all patients underwent antegrade subchondral drilling. Autologous conditioned plasma (ACP) was prepared according to the manufacturer’s protocol (Arthrex, Naples, FL, USA), and thrombin was generated using the Thrombinator™ system, Arthrex, Naples, FL, USA. The harvested cartilage was minced to a homogeneous paste-like consistency and mixed with ACP prior to implantation. Autologous thrombin and a thrombin–ACP mixture were then applied to stabilize the graft and stabilize the cartilage graft with autologous fibrin. The lateral ankle ligaments were thoroughly evaluated and treated. ATFL and CFL stumps were identified and exposed. One corkscrew anchor (3.5 mm) was inserted into the fibula. The ATFL and/or CFL were then tensioned with the ankle in slight eversion and securely fixed to the fibula. The ligament remnants were carefully identified, mobilized, and prepared prior to fixation to allow anatomical reattachment at their fibular footprint. To reinforce the repair, a retinacular reinforcement (Gould modification) [12] was mobilized and imbricated and sutured to the distal fibula based on the publication by Tourné et al. [13] Postoperatively, the ankle was immobilized in a neutral position using a dorsal splint for a duration of 6 weeks during the rehabilitation phase. All the surgeries were performed by one surgeon.

### 2.2. Clinical Evaluation

A functional assessment was carried out via questionnaire (FFI score), clinical assessment (VAS), return-to-sport test, and motor-cognitive test (Skillcourt technology) [14]. The Motor-cognitive testing (Skillcourt system) includes a random star run test, a left and right test, a single leg stand, a single leg squat and a single leg stability test [15]. A clinical examination, including subjective functional and physical examinations, was performed by an experienced orthopedic surgeon.

### 2.3. Skillcourt System

The Skillcourt tests are defined as “motor-cognitive” paradigms in accordance with previous studies that have assessed similar task. These tests place higher motor-cognitive demands compared to computer-based cognitive assessments, as even simple motor challenges, such as walking or maintaining balance, can induce dual-task interference [16] when combined with cognitive tasks [14,16]. All tests were conducted on a Skillcourt system with a 5 × 5 m rubber mat area. For the dynamic agility assessments, the eight square target zones (four corners, four sides) at the edges of the Skillcourt area were used [15]. All measurements were repeated three times.

### 2.4. Random Star Run Test

To assess agility performance, a Reactive Agility Test (RAT) [14] was conducted. In this version of the Random Star Run, the target fields illuminated in a random order, thereby introducing a decision-making component. This required the participants to perceive and process visual stimuli and modulate their motor output. Participants were instructed to complete the task as quickly as possible [15].

Procedure:

Starting Position: The participant begins in an athletic ready position at the center of the Skillcourt field.

Task: After a 3 s countdown, one of the eight target fields lights up. The participant must run to the corresponding target, step on it with at least one foot, and return to the center field by changing direction.

Measurement: The system records the time it takes to reach each target, the accuracy of the movements, and the participant’s reaction time [15].

### 2.5. Left and Right Test

In the Left’n Right test, participants are required to reach fields to the left or right of the starting area as quickly as possible, in a randomized order, and tap them with their feet [14].

Procedure:

Starting Position: The participant begins in an active stance at the center of the Skillcourt field.

Task: After a signal, one of the target zones, either to the left or right of the center, lights up randomly. The participant must quickly run to the illuminated target and tap it with their foot.

Measurement: The system records the response time, accuracy in reaching the target, and the speed of movement [15].

### 2.6. Single-Leg Stand Test

The Single-Leg Stand Test on the Skillcourt system is designed to assess balance and stability. In this test, the participant is required to stand on one leg in the center of the Skillcourt field while maintaining their balance for a predetermined period of time. The test may involve additional cognitive or sensory challenges depending on the specific protocol being used. This test is commonly used to evaluate static balance, proprioception, and the participant’s ability to maintain stability under controlled but potentially disruptive conditions. It can be part of a broader motor–cognitive assessment, combining balance with reaction time and decision-making tasks [15].

Procedure:

Starting Position: The participant stands on one leg at the center of the Skillcourt field, keeping the other leg raised.

Task: The participant must maintain balance on the supporting leg for a specified duration while visual stimuli (e.g., lit targets) appear on the Skillcourt system. The participant may be required to respond by interacting with the targets.

Measurement: The system quantifies the center-of-pressure (CoP) stability duration the participant maintains balance, the accuracy of target interactions, and the participant’s ability to focus on visual cues while balancing [15].

### 2.7. Single-Leg Squat Test

The Single Leg Squat Test on the Skillcourt system assesses lower limb stability, motor control, and proprioception. During the test, participants are required to perform a squat on one leg while maintaining balance and control [15].

Procedure:

Starting Position: The participant stands on one leg in the center of the Skillcourt field, with the other leg raised.

Task: The participant performs a controlled squat on the supporting leg, maintaining proper lower-limb alignment. The movement may be guided by visual targets on the Skillcourt system.

Measurement: The test evaluates the participant’s ability to maintain postural stability while executing the squat and responding to cognitive stimuli, such as reaction time and accuracy [15].

### 2.8. Single Leg Stability Test

The Single Leg Stability Test on the Skillcourt system is designed to evaluate balance, proprioception, and motor control while standing on one leg. The test combines static balance with dynamic responses to visual or auditory cues [15].

Procedure:

Starting Position: The participant stands on one leg at the center of the Skillcourt field, maintaining balance.

Task: The participant must react to visual (e.g., lit targets) or auditory stimuli by performing a specified movement, such as tapping a target or adjusting posture, all while maintaining balance on the supporting leg.

Measurement: The test assesses the duration of balance maintenance, reaction speed, and accuracy in responding to the stimuli [15].

### 2.9. Statistical Analysis

All data are presented as mean and standard deviation. Postoperative results of skill court measurements were compared using Student *t*-test to compare paired variables. Statistical analyses were performed using the SPSS software package (version 29; 134 IBM SPSS Statistics, Chicago, IL, USA). A two-sided significance level of *p* < 0.05 was used to determine statistical significance.

## 3. Results

A total of 9 patients with lateral osteochondral lesions and concomitant chronic instability were included in this study. The average age was 39.8 years. The average follow-up was 15.8 months (minimum 9 months; maximum 23 months) (Table 2). Among them, 5 patients had an osteochondral lesion of the talus on the left side, and 4 patients had an osteochondral lesion on the right side. No major postoperative complications were observed. All patients were satisfied with the postoperative result. The Foot Function Index showed that none of the patients experienced any restrictions in everyday life postoperatively (FFI: 14.92, SD 12.74). On physical examination, mechanical stability was restored in all patients and no patient complained of ankle instability at the time of follow-up. Seven patients reported participating in regular athletic activity. Of these, 71.4% (5/7) returned to sport at the same level as before the injury. All patients were satisfied with the surgery. Motor-cognitive assessment with the Skillcourt system was performed at the final follow-up. The average postoperative VAS score during all Skillcourt system exercises was 1.42 (SD = 1.62).


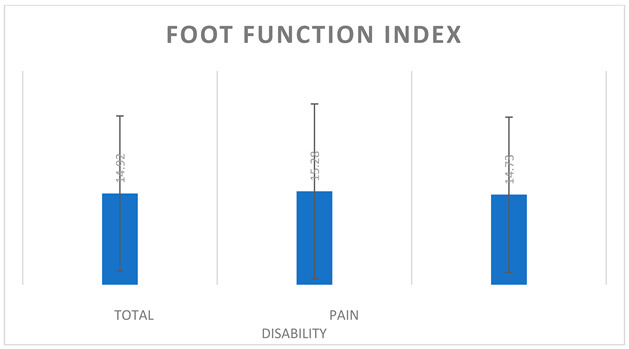


### 3.1. Random Star Run Test

When comparing the time required to reach fields on the left or right side of the training area, there is no significant difference between the operated and non-operated side. Similarly, there was no significant difference in the contact time with the individual pitches on the right or left side of the training area between the operated and non-operated side.

### 3.2. Left and Right Test

The evaluation showed that no significant difference could be determined for the contact times with the respective fields, whether the fields were located towards the operated leg or on the opposite side.

### 3.3. Single Leg Stand Test

There is no statistically significant difference between the symmetry scores of the operated and non-operated sides.

### 3.4. Single Leg Squat Test

Only 5 patients could be included in this test. A total of 4 patients were unable to perform this test due to insufficient dorsiflexion in the operated ankle joint. In the 5 patients included, there was no significant difference between the operated and non-operated side.

### 3.5. Single Leg Stability Test

This skillcourt test revealed a statistically significant difference between the anterior region of the operated and non-operated leg (*p* = 0.027). According to Cohen’s d test, a large effect size can be assumed (d = 0.9). The posterior medial and lateral areas showed no difference between the operated and non-operated side.

## 4. Discussion

This study shows that the simultaneous management of lateral OLT and concomitant CAI leads to satisfactory clinical results after an average of 15.8 months. There were no postoperative complications in any of the patients. The ligament reconstruction technique augmented by inferior extensor retinaculum reinforcement, as described by Tourné et al. [13], was used in all patients in this study. This technique is particularly relevant in the treatment of chronic lateral ankle instability (CAI), especially when combined with lateral osteochondral lesions of the talus (OLT), which are more often of traumatic origin and associated with ligamentous insufficiency [5]. The limited dorsiflexion of the upper ankle joint, which is not noticeable in the patient’s everyday life and during normal sporting activity, was determined during the cognitive-motor examination of the patients using the Skillcourt system. There was a significant difference between the operated and non-operated leg in the single leg stability test and in 5 patients a restriction in the performance of the single leg squat due to the reduced dorsiflexion. Although no clinical instability was detected at follow-up, the observed difference in the single-leg stability test likely reflects subtle functional differences in motor control rather than residual mechanical laxity. In the study by Cao et al., a reduced static and dynamic sagittal range of motion of the ankle joint was observed in patients with osteochondral lesions and chronic instability [17]. However, the main finding here was limited plantar flexion in the patients. Although passive clinical measurements showed no significant restriction of dorsiflexion compared to the healthy limb, the Single-Leg Squat test revealed functional limitations in nearly half of the patients (*n* = 4). This discrepancy suggests that standard non-weight-bearing assessments may fail to detect dynamic restrictions that only become apparent during more demanding functional tasks. Consequently, these findings emphasize the importance of incorporating dynamic assessments to identify subtle kinetic chain compensations that isolated measurements might overlook.

Simultaneous surgical treatment of the ostechondral lesion and chronic instability does not lead to a worsening of the postoperative outcome [18]. In contrast to patients with chronic instability alone, patients with concomitant chronic instability and osteochondral lesion have a reduced preoperative sagittal range of motion of the ankle joint [17,19]. Combined procedures result in improved joint stability and favorable clinical results [18]. The retinacular augmentation (The Gould-Broström modification) has been shown to enhance biomechanical stability and reduce recurrence rates of instability, especially in high-demand patients [20]. In our series, an open approach was chosen because the ligament reconstruction technique described by Tourné et al. allows direct visualization of the lateral ligament complex and facilitates precise anatomical reconstruction, whereas arthroscopic techniques are primarily limited to anchor-based repairs. However, the potential for minor postoperative motion restrictions warrants further investigation.

All previous studies, though, have included lateral and medial lesions of the talus. As is already known, lateral osteochondral lesions in particular are more frequently associated with chronic instability of the ankle joint than medial osteochondral lesions of the talus [21]. Thus, the comparability of our study with existing studies is limited because our study only included patients with osteochondral lesions and concomitant chronic instability. In a recent systematic review by Anwander et al., it was again shown that bone marrow stimulation (BMS) is used regularly, especially for smaller lesions (<100 mm^2^) without bone defects [22]. However, numerous invasive treatment options have been described for larger lesions (>100 mm^2^), but there is no consensus to date [3,23].

In contrast to the study by Ackermann et al., which included osteochondral lesions (OLT) of both the medial and lateral talar shoulder with concomitant ankle instability treated via AMIC and, if necessary, lateral ligament stabilization, the present investigation focused only on osteochondral lesions of the lateral talar shoulder [24]. Notably, the defect size in our cohort was smaller, with an average area of 0.65 cm^2^, and all patients underwent AutoCart cartilage transplantation. Despite the smaller defect size and specific localization, our findings align with those of Ackermann et al., confirming that the combination of biological cartilage repair and mechanical stabilization yields favorable outcomes. These results emphasize that, even in smaller lateral lesions, addressing both the osteochondral defect and accompanying ligamentous instability remains essential for optimal joint function and patient satisfaction. Although biologically augmented cartilage repair is typically recommended for larger lesions, the open approach required for lateral ligament reconstruction in our patients provided direct access to the defect, allowing cartilage restoration to be performed without iatrogenic morbidity.

Although clinical evidence remains limited, several recent case series have reported encouraging short-term outcomes after minced cartilage implantation for OLT. Two publications on minced cartilage as technical notes, but without clinical data. A recent publication retrospectively examined 25 patients who were treated with minced cartilage [25]. Three groups were differentiated in this study, cartilage mincing with fibrin fixation, cartilage mincing with the use of a membrane and fibrin fixation and cartilage mincing combined with the “AutoCart™” procedure. The minced cartilage combined with AutoCart™ procedure group included 9 patients with an average defect size of 102.8 mm^2^ ± 72.3 mm^2^ [25]. In 5 cases, revision surgery from the minced cartilage combined with the “AutoCart™” procedure was performed. According to this study, the complication rate depends on the fixation method and only remained low with fibrin fixation. Patients with minced cartilage combined with the “AutoCart™” procedure without fibrin fixation showed a high revision rate (56%). In the study by Kühle et al., the complication rate appeared to depend on the fixation technique, with higher revision rates reported in cases without fibrin fixation [25]. These findings highlight the potential importance of adequate graft stabilization when using minced cartilage techniques. In our study, no complications were observed during the follow-up period. All patients were satisfied postoperatively and 55.6% of patients returned to their pre-injury sports level. We used minced cartilage enriched with ACP, which was stabilized with an autologous thrombin-enriched fibrin clot. The average size of the osteochondral lesion of the lateral talus in our study was 64.8 mm^2^ ± 30.2 mm^2^. Compared to the cohort in the study by Kühle et al., our patient population exhibited a smaller osteochondral defect. Consequently, the two studies are not directly comparable [25].

Williamson et al. recently reported encouraging short-term outcomes following autologous minced cartilage implantation for osteochondral lesions of the talus, demonstrating significant improvements in pain and functional outcome scores at one-year follow-up. These findings support the concept of minced cartilage as a viable single-step cartilage repair technique for talar lesions, although the current clinical evidence remains limited and further studies with larger cohorts and longer follow-up are required to confirm long-term durability [26].

The functional outcomes in our cohort, as measured by the Foot Function Index (FFI), were comparable to those reported in previous studies. In our study, the mean postoperative FFI was 14.92 (SD 12.74), indicating low levels of functional impairment. Similar improvements in FFI scores following treatment of osteochondral lesions of the talus were described in the systematic review by Walther et al. (2021), in which three studies reported postoperative FFI values in a comparable range (Walther et al. 2013 FFI: 28, Gottschalk et al. 2017 FFI: 24, Kubosch et al. 2015 FFI: 34) [27,28,29,30]. These findings indicate that the functional outcomes in our cohort are similar to those reported in previous studies on the treatment of osteochondral lesions in the ankle.

In summary, it can be concluded that the combination of minced cartilage grafting and subchondral bone stimulation could represent a therapeutic option for osteochondral lesions < 100 mm^2^ of the lateral talus. However, there is currently still a lack of clinical data for a conclusive assessment.

### Limitations

Given the retrospective design and the small sample size, the present study should primarily be interpreted as a preliminary exploratory case series evaluating the feasibility and short-term functional outcomes of combined minced cartilage implantation and ligament reconstruction. This study has several limitations. First, this study has a retrospective design, and it is a report of a case series without any comparison between different treatment modalities. Second, no mid-term or long-term outcomes were evaluated. There was a follow-up of 15.8 months. Therefore, no statements on medium- to long-term postoperative results can be made. Furthermore, we are aware that cysts can develop in the talus as a result of the drilling. We did not routinely perform an MRI examination postoperatively. Therefore, we cannot make any statements about MR-graphic cartilage regeneration and regeneration of the subchondral bone. Larger prospective studies with medium and long-term follow-ups are required to conclusively evaluate the clinical role of this new method in modern cartilage surgery for the foot and ankle. Due to the retrospective design, preoperative patient-reported outcome measures (PROMs) were unavailable, limiting the ability to objectively assess pre- to postoperative improvement. In addition, detailed return-to-sport parameters were not consistently documented due to the retrospective study design, limiting the assessment of postoperative functional recovery. The short follow-up period represents a limitation, as long-term evaluation is crucial in cartilage repair and ligament surgery to determine durability and recurrence rates. Another limitation is that the mean lesion size in our cohort was below the threshold of 100 mm^2^ that is commonly suggested for biologically augmented cartilage repair procedures. Furthermore, future studies including a comparison group of patients with isolated chronic ankle instability without osteochondral lesions would be valuable for better determining the specific contribution of cartilage repair to clinical outcomes.

## 5. Conclusions

The combined treatment of lateral osteochondral lesions of the talus and chronic ankle instability using autologous minced cartilage implantation and ligament reconstruction with retinaculum augmentation yields favorable short-term clinical and functional outcomes. In this cohort, no postoperative complications were observed, and patients demonstrated high satisfaction with minimal functional impairment.

Although subtle deficits in demanding motor-cognitive tests were detected, these findings likely reflect minor alterations in neuromuscular control rather than residual mechanical laxity. The open ligament reconstruction technique with retinacular augmentation provides reliable stabilization and enables concomitant cartilage restoration of the osteochondral defect without additional surgical morbidity.

These preliminary findings support the technical feasibility and short-term clinical applicability of combining biological cartilage repair with mechanical stabilization for lateral talar lesions < 100 mm^2^. However, given the limited sample size, retrospective design, and short follow-up, further prospective studies with larger cohorts and long-term evaluation are required to confirm survivorship and define the role of this technique in comparison to established treatment options.

## Figures and Tables

**Table 1:** Clinical characteristics, diagnoses, and additional treatments of patients with chronic lateral ankle instability and osteochondral lesions.

	Trauma	Regular Athletic Activity/Return to Sport	Diagnosis	Additional Treatment	Previous Surgery
Patient 1	ankle sprain	yes/yes	Osteochondral lesion of the lateral talus with early-stage osteoarthritis and chronic lateral ankle instability (Status post retrograde drilling)	Cyst filling with cancellous bone from the calcaneus	Arthroscopy with retrograde drilling
Patient 2	ankle sprain	yes/yes	Chronic lateral ankle instability and osteochondral lesion of the lateral talus, tibia and syndesmotic lesion	Syndesmosis reconstruction with tight-rope	no
Patient 3	ankle sprain	no/no	Chronic lateral ankle instability and osteochondral lesion of the lateral talus and tibia, Intra-articular loose body	Resection of the loose body	no
Patient 4	ankle sprain	yes/no	Osteochondral lesion of the lateral talus with early-stage osteoarthritis and chronic lateral ankle instability	Cheilectomy, Cyst filling with cancellous bone from the calcaneus	no
Patient 5	ankle sprain	yes/yes	Chronic lateral ankle instability and osteochondral lesion of the lateral talus, Intra-articular loose body	Resection of the loose body	no
Patient 6	ankle sprain	yes/yes	Chronic lateral ankle instability and osteochondral lesion of the lateral talus	Cheilectomy calcaneocuboideal joint and Tenosynovectomy of the peroneal tendons	no
Patient 7	ankle sprain	yes/no	Chronic lateral ankle instability and osteochondral lesion of the medial and lateral talus, tendosynovitis of the peroneal tendons	/	no
Patient 8	ankle sprain	yes/yes	Chronic lateral ankle instability and osteochondral lesion of the lateral talus	/	no
Patient 9	ankle sprain	yes/yes	Chronic lateral ankle instability and osteochondral lesion of the lateral talus and tibia	Cheilectomy	

**Table 2:** Patients Characteristics.

	*n = 9 Patients*
*Age (mean)*	*39.8 years*
*Follow-up (mean)*	*15.2 months*
*Female sex*	*3*
*Lesion size mm^2^ (mean)*	*64.8 mm^2^ ± 30.2 mm^2^*
*Time to surgery (mean)*	*16.6 months*
*Previous surgeries*	*1/ 9 patients (11.1%)*

## Data Availability

The original contributions presented in this study are included in the article. Further inquiries can be directed to the corresponding author.
